# Structure of Alcohol Oxidase from *Pichia pastoris* by Cryo-Electron Microscopy

**DOI:** 10.1371/journal.pone.0159476

**Published:** 2016-07-26

**Authors:** Janet Vonck, David N. Parcej, Deryck J. Mills

**Affiliations:** Department of Structural Biology, Max Planck Institute of Biophysics, Frankfurt am Main, Germany; Instituto de Tecnologica Química e Biológica, UNL, PORTUGAL

## Abstract

The first step in methanol metabolism in methylotrophic yeasts, the oxidation of methanol and higher alcohols with molecular oxygen to formaldehyde and hydrogen peroxide, is catalysed by alcohol oxidase (AOX), a 600-kDa homo-octamer containing eight FAD cofactors. When these yeasts are grown with methanol as the carbon source, AOX forms large crystalline arrays in peroxisomes. We determined the structure of AOX by cryo-electron microscopy at a resolution of 3.4 Å. All residues of the 662-amino acid polypeptide as well as the FAD are well resolved. AOX shows high structural homology to other members of the GMC family of oxidoreductases, which share a conserved FAD binding domain, but have different substrate specificities. The preference of AOX for small alcohols is explained by the presence of conserved bulky aromatic residues near the active site. Compared to the other GMC enzymes, AOX contains a large number of amino acid inserts, the longest being 75 residues. These segments are found at the periphery of the monomer and make extensive inter-subunit contacts which are responsible for the very stable octamer. A short surface helix forms contacts between two octamers, explaining the tendency of AOX to form crystals in the peroxisomes.

## Introduction

Methylotrophic yeasts can utilize methanol as sole carbon source. The first step of methanol metabolism, the oxidation with molecular oxygen to formaldehyde and hydrogen peroxide, is catalysed by alcohol oxidase (AOX) (EC 1.1.3.13) and occurs in specialized organelles, the peroxisomes, where H_2_O_2_ is decomposed by catalase. AOX is a member of the GMC (glucose–methanol–choline) oxidoreductase family [1–3] and uses a non-covalently bound FAD cofactor. AOX catalyses the oxidation of small primary alcohols [4] using a two-step mechanism, first a reductive half-reaction, where the substrate is oxidised while the FAD is reduced to FADH_2_, followed by an oxidative half-reaction, where the flavin is reoxidized by O_2_ and H_2_O_2_ is produced [5, 6].

AOX has a low affinity for methanol and oxygen [4, 7], and when the yeasts are grown on methanol as sole carbon and energy source, the synthesis of large amounts of AOX results [7][8]. This is driven at the transcriptional level by the exceptionally strong AOX promoter. The promoter is also tightly regulated, being repressed by alternative carbon sources as well as activated by methanol. This has made *Pichia pastoris* a popular system for heterologous protein production, where the foreign gene is placed under control of the AOX promoter [9]. The amount of AOX protein produced in response to methanol is so high as to form large crystalloids in the peroxisomes, which under these conditions can occupy up to 80% of the cell volume [10]. Many other proteins are present inside the peroxisome, including (in addition to catalase) the key enzymes involved in methanol assimilation. One of these proteins, dihydroxyacetone synthase (DHAS), which converts formaldehyde and xylulose-5-phosphate to glyceraldehydephosphate and dihydroxyacetone, also occurs in large amounts [11, 12], but the peroxisomal crystalloids have been shown to consist of AOX alone and they can be formed readily *in vitro* from the isolated protein [13, 14]. The crystalloids have an open structure with large holes, where presumably the other proteins are located, allowing catalase and DHAS fast access to their substrates, H_2_O_2_ and formaldehyde, respectively [14].

Although the peroxisomal crystal form of AOX can be easily grown *in vitro*, these crystals do not diffract X-rays to high enough resolution to determine the structure [13, 15]. AOX in a different crystal form, grown in the presence of azide, diffracted to 2.7 Å resolution [16], but although the presence and orientation of the octamers in the asymmetric unit was established by electron microscopy [17], the structure was not solved until very recently [18]. Crystal structures are available for several other members of the GMC family: choline oxidase (CHOX) [19][20][21], aryl alcohol oxidase (AAOX) [3], glucose oxidase (GOX) [22, 23], pyranose 2-oxidase [24–26], cellobiose dehydrogenase [27], cholesterol oxidase [28][29], and an hydroxynitrile lyase related to aryl alcohol oxidase [30]. All these proteins adopt a Rossmann-fold in their FAD-binding domain [31] and share a domain structure. The oligomeric state of the proteins in the GMC family is varied. While most function as monomers, glucose oxidase is a dimer, pyranose 2-oxidase a tetramer, and AOX an octamer [4, 32]. AOX is with 663 amino acids larger than any of the other proteins, mainly due to a more than 70 residues long insert near the C-terminus that is thought to be involved in oligomerisation.

The recent introduction of direct electron detectors in cryo-electron microscopy (cryo-EM) [33] has now made it possible to determine the structure of macromolecular complexes at near-atomic resolution using this technique [34–41]. Here we present a cryo-EM map of *Pichia pastoris* alcohol oxidase at a resolution of 3.4 Å, which has allowed us to determine the structure of this protein.

## Results and Discussion

### Structure determination

AOX was purified from *Pichia pastoris* cells that had been grown overnight on methanol as carbon source following procedures described previously and cryo-EM images were taken on a JEOL 3200 FSC electron microscope with a K2 direct electron detector in movie mode. The movie frames were aligned before image processing to correct for beam-induced particle motion [35].

**Fig 1:**
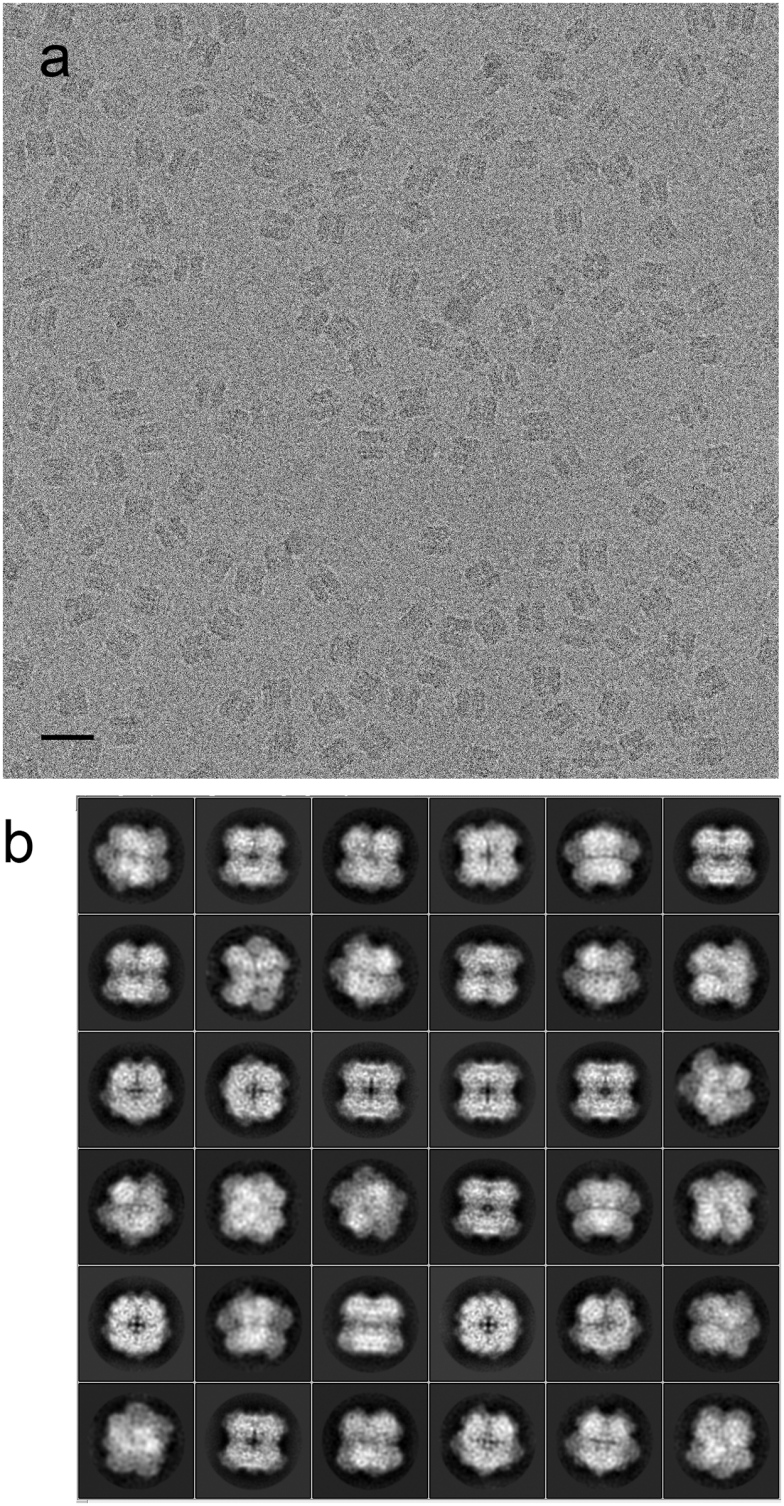
Cryo-electron microscopy of AOX. (a) Micrograph of AOX collected on a JEOL 3200 FSC electron microscope using a K2 direct electron detector. The defocus was determined as 1100 nm. The scale bar represents 20 nm. (b) 2D class averages calculated using Relion show the octameric particle in different orientations.

The sample is pure and monodisperse; the 600-kDa AOX particles are clearly visible on the K2 images, even at defocus values well below 1 μm (Fig 1a). 2D classification shows that the particles are present in different orientations in the ice, with a majority displaying top views with clear fourfold symmetry as well as side views (Fig 1b), supporting an octameric structure with D4 symmetry.

**Fig 2:**
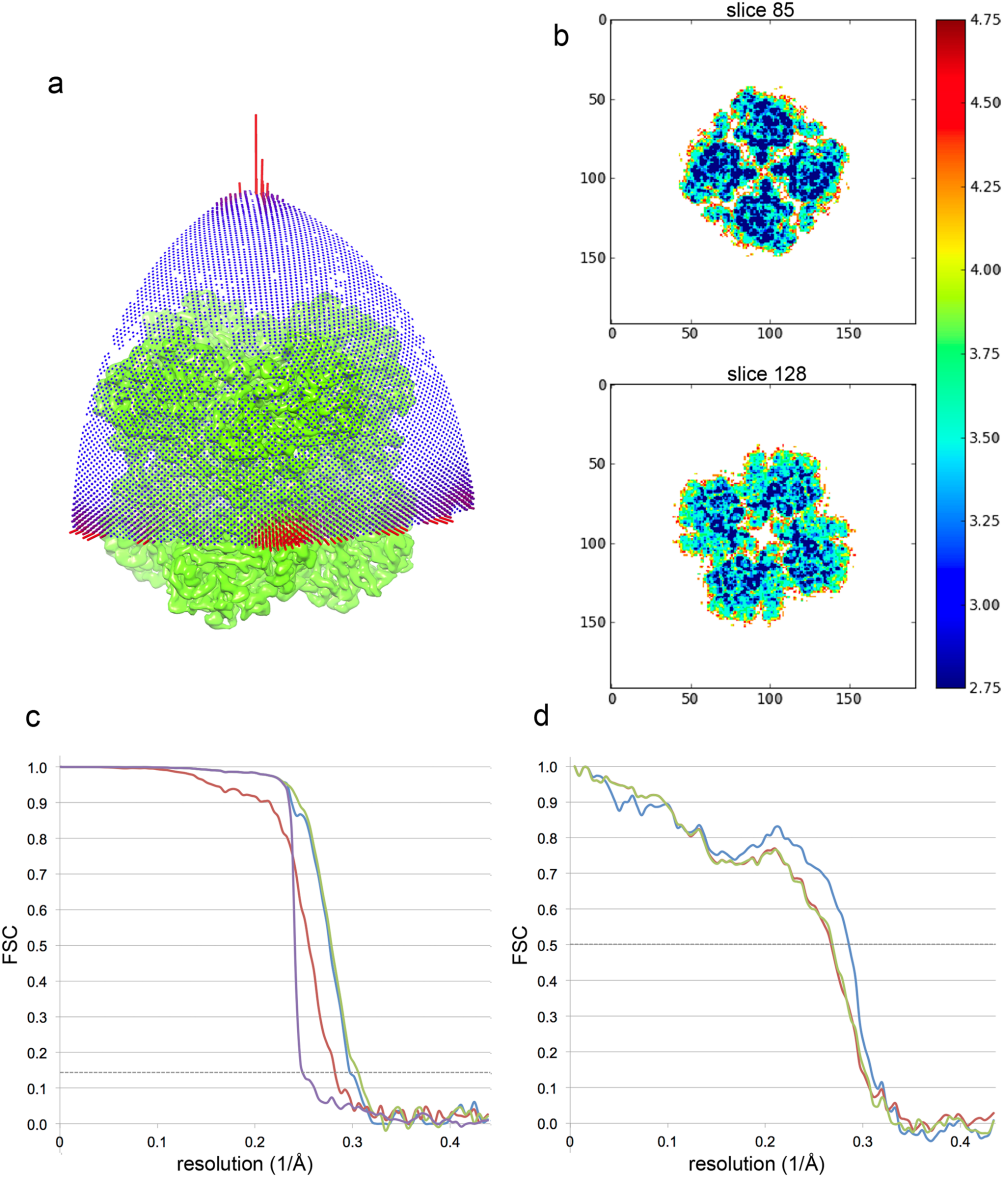
Map quality and validation of map and model. (a) Orientation distribution of the particles. One asymmetric unit of the D4 symmetric map is shown. The height of the cylinders is proportional to the number of particles in the orientation indicated. In green the unsharpened final map is shown. Top views along the 4-fold axis (top) and side views (bottom) are overrepresented in the dataset. (b) Local resolution analysis by ResMap [45] shows that the complex is rigid without flexible parts. (c) Gold-standard FSC of the final map after masking as determined by the post-processing procedure in RELION [44] indicates a resolution of 3.37 Å. A B factor of −147 Å^2^ was determined and applied to sharpen the map. (d) FSC curves between the model and the cryo-EM map. The FSC curve between the final refined model and the reconstruction from all particles (blue) indicates a resolution of 3.5 Å. FSC between the model refined in the reconstruction from half the particles and the reconstruction from that same half (FSCwork, red), and between this model and the reconstruction from the other half of the particles (FSCfree, green) both indicate the same resolution (3.7 Å), showing that no overfitting of the model to the map has taken place.

An initial 3D model was created from the 2D class averages, applying D4 symmetry. 3D classification in Relion with this as starting model yielded consistently a single good class, containing >85% of the particles. Although no symmetry was applied during this step, the reconstruction displayed clear D4 symmetry. This symmetry was thus applied during refinement using the gold standard method in Relion [42]. Although as mentioned above and evident from the 2D class averages, the particles adopted preferred orientations in the ice, all orientations were represented in the reconstruction (Fig 2a). The final map calculated using 50,000 particles from 371 micrographs had a resolution of 3.5 Å. Applying the local frame alignment protocol [43] improved the resolution to 3.4 Å as determined by the post-processing procedure in Relion [44].

**Fig 3:**
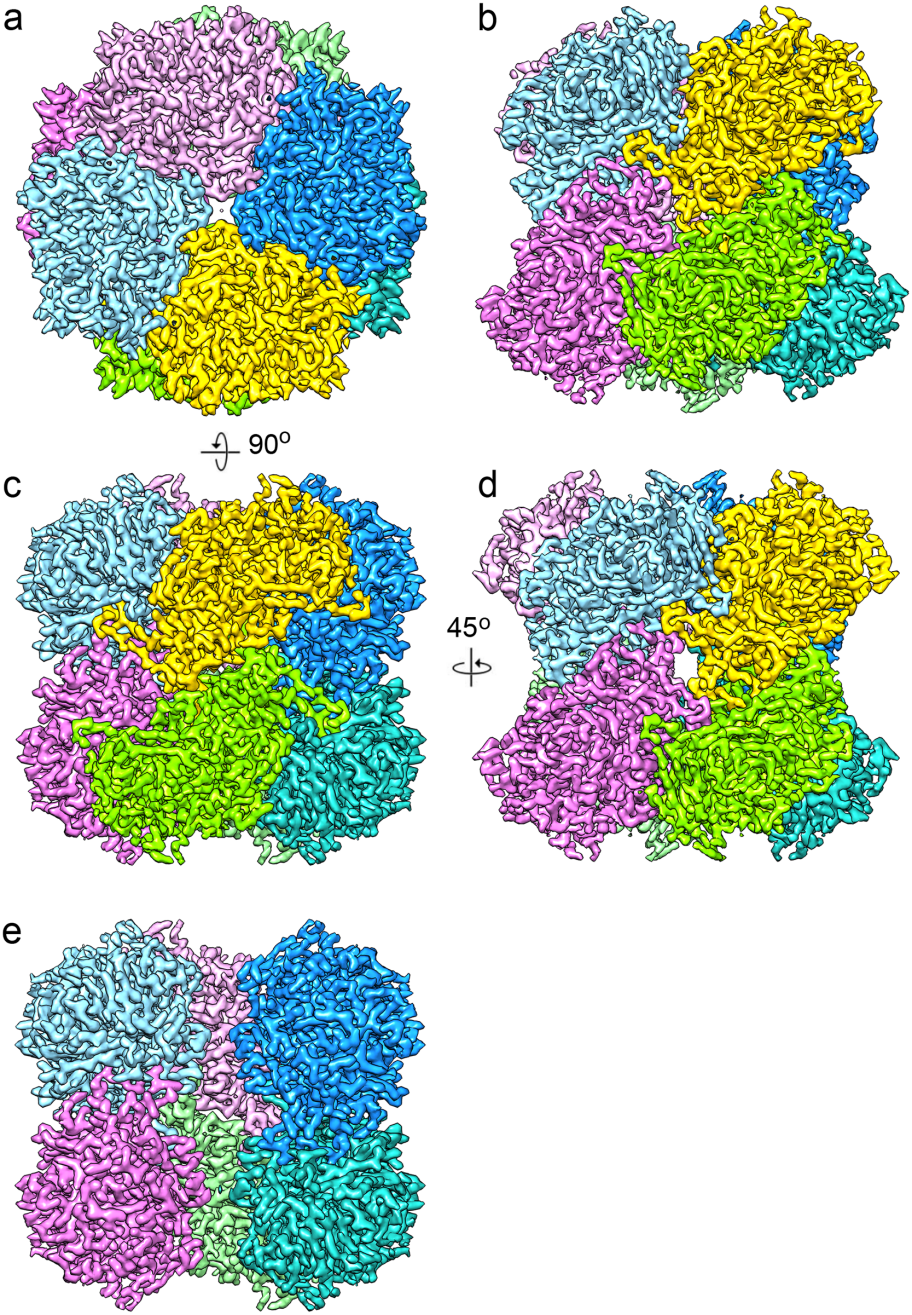
3.4 Å cryo-EM map of AOX. The map was sharpened by a negative B-factor of -147 Å^2^. Each of the eight monomers is shown in a different colour. (a) Top view along the fourfold axis. (b) View in the most common orientation. (c) Side view along a twofold axis. (d) Side view along another twofold axis. (e) Same view as (c), with the front two monomers removed to show the interior of the octamer.

**Fig 4:**
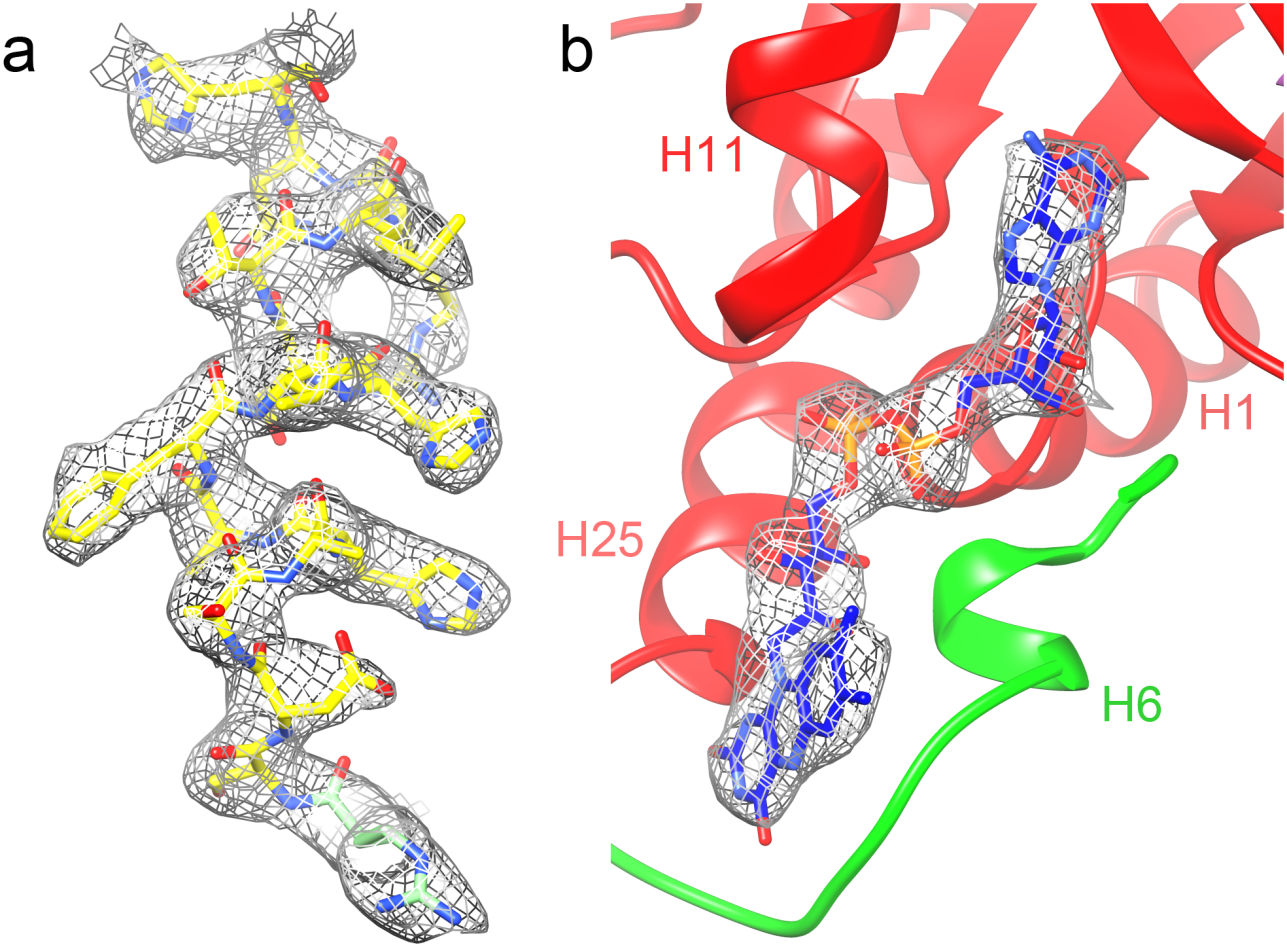
Details of the 3.4 Å map. (a) α-helix H10 with model fitted. (b) The FAD cofactor and its binding pocket. Domain colors as in Fig 5a.

The map at 3.4 Å resolution (Fig 3) shows clear α-helices and β-sheets and side chain densities for most residues (Fig 4, S1 Movie). Local resolution analysis by ResMap [45] (Fig 2b) gives no indication of flexible regions, and only the surface of the protein and the subunit interfaces show worse than 4 Å resolution. In order to fit the AOX residues to the map, advantage was taken of the structures available for other members of the GMC family. While the structures differ considerably, many core elements are conserved and the quality of the map and visibility of side chains made it easy to fit the sequence correctly. The FAD density was also well resolved and indicated an FAD conformation typical for the GMC family (Fig 4b). Structural elements unique for AOX are mostly located in the C-terminal half of the protein and include, apart from some short sections, a loop (364–392) including two α-helices connecting two β-strands of the 5-stranded parallel sheet (see below), a 75-residue insert (477–551) and a ~25 residue C-terminal extension (637–663) (S1 Fig). These inserts were built *de novo* into the density. Apart from the N-terminal methionine, which is apparently absent in the mature enzyme [4, 8], the whole chain could be traced with the large majority of side chains showing clear density (Fig 4). Notable exceptions are the highly radiation-sensitive carboxylate side chains of glutamate and aspartate [37, 46] and surface-exposed side chains, mostly of lysines and a few arginines. Comparison with the very recent 2.35-Å crystal structure of AOX [18] (pdb 5hsa) shows a high correspondence between the two models with an RMSD of 0.58 Å for the C-α atoms and 1.12 Å for all atoms.

**Fig 5:**
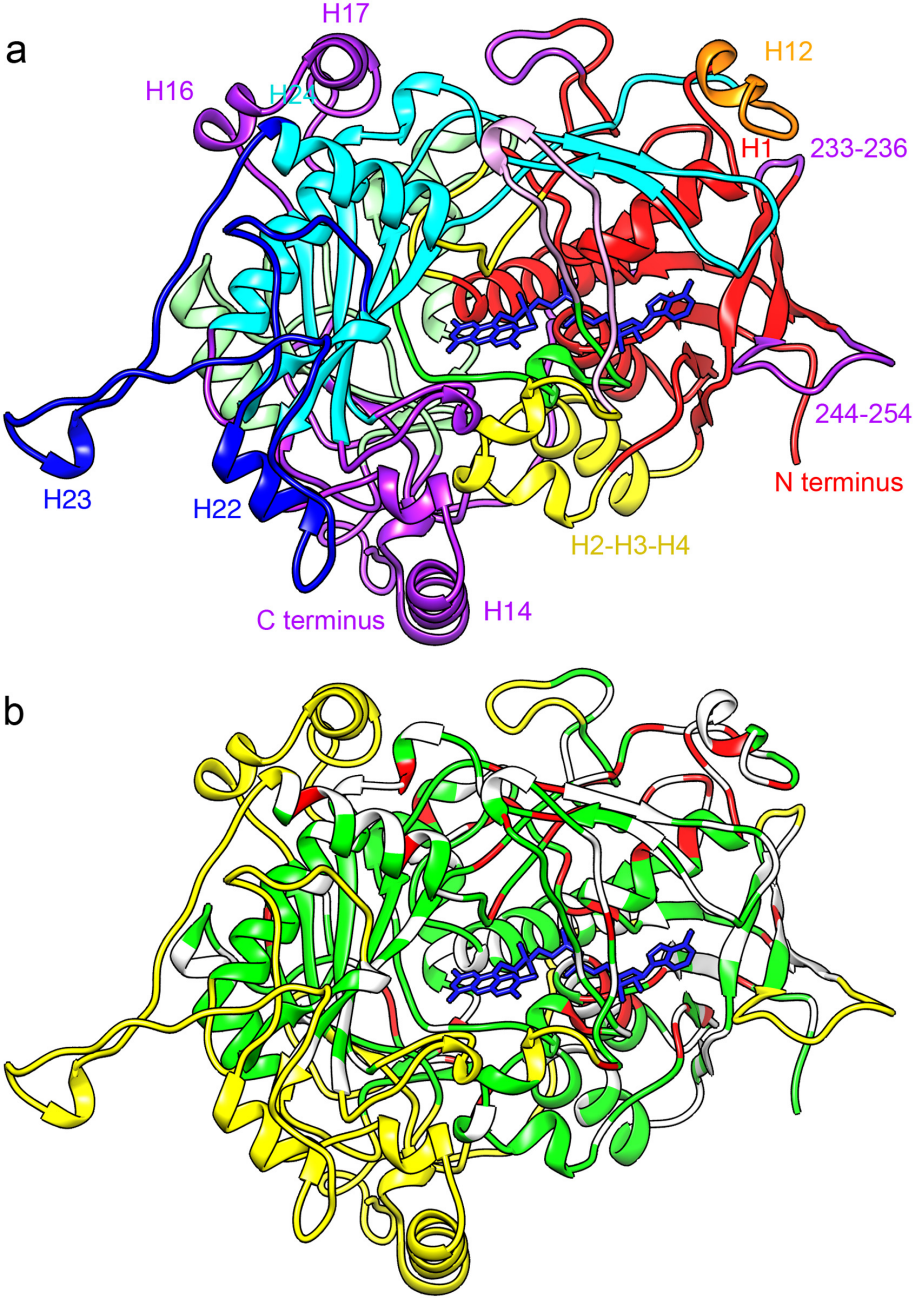
Model of the AOX monomer. (a) Structural domains. The domain assignment for the GMC family [2]) is as follows: FAD-binding domain (red); FAD covering lid (pink); extended FAD-binding domain (yellow); flavin attachment loop (green) and intermediate region (light green); substrate-binding domain (cyan). Helix H12, which forms crystal contacts, is shown in orange. The longest AOX-specific insert 481–548 is shown in dark blue, other AOX-specific sequences are colored purple. The FAD cofactor is blue. The location of the five β-sheets A-E and some α-helices (nomenclature from S1 Fig) and sequence mentioned in the text is indicated. The view of the monomer is along the fourfold axis from inside the complex. (b) Conservation of residues. Residues conserved in the GMC family (see S1 Fig) are shown in red and residues conserved in AOX are green; unconserved residues are white. Structural elements unique for AOX are yellow. Note that the FAD binding domain (right; red in (a)) has high conservation in the GMC family, while the substrate binding domain centered around β-sheet C (left; cyan in (a)) is conserved between AOX species.

**Fig 6:**
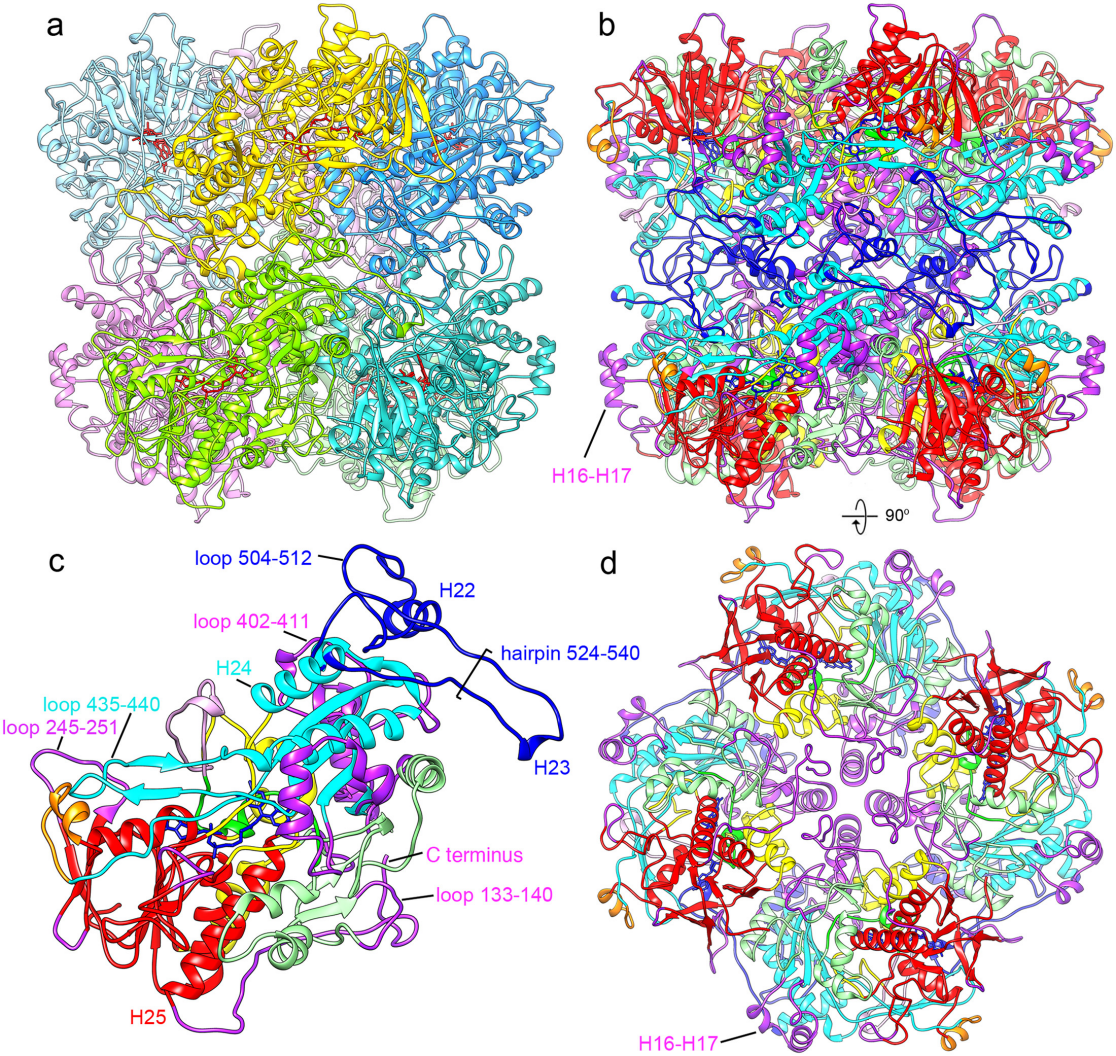
Intersubunit contacts. The octamer is shown in (a) with every subunit in a different color and in (b) in the same orientation with domain colors as in Fig 5a. One monomer (the green one in (a)) is shown in (c) with the location of residues discussed in the text indicated. The interface of two tetrameric rings is almost exclusively made of the AOX-specific insert 481–548 (dark blue); the only exception is the dimer contact between the loops 408–409. (d) A view rotated 90° relative to (b). Only one tetrameric ring is shown for clarity. The C-terminal AOX-unique tail (purple) extends from the long helix H25 (red) of the FAD binding domain. The four copies form a ring around the fourfold axis and the last four amino acids are buried inside the complex.

The AOX monomer (Fig 5) has 18 β-strands in five sheets (S1 Fig, S2 Movie). All β-sheets are conserved in the GMC family; they include the 5-stranded parallel sheet and the 3-stranded antiparallel sheet of the FAD-binding Rossmann fold (sheet A and D), a 6-stranded antiparallel sheet forming the substrate binding domain (sheet C), and two 2-stranded sheets (B and E). The structure has 25 helices, five of which occur in the AOX-specific insertion elements. The core structure is very similar to that of the other GMC family members and the FAD-binding and substrate-binding domains are clearly recognisable (Fig 5a). The AOX-specific inserts are located at the periphery of the monomer, where they mediate the inter-subunit contacts and form a tightly packed oligomer (S3 and S4 Movies, Fig 6).

### The FAD binding domain

**Fig 7:**
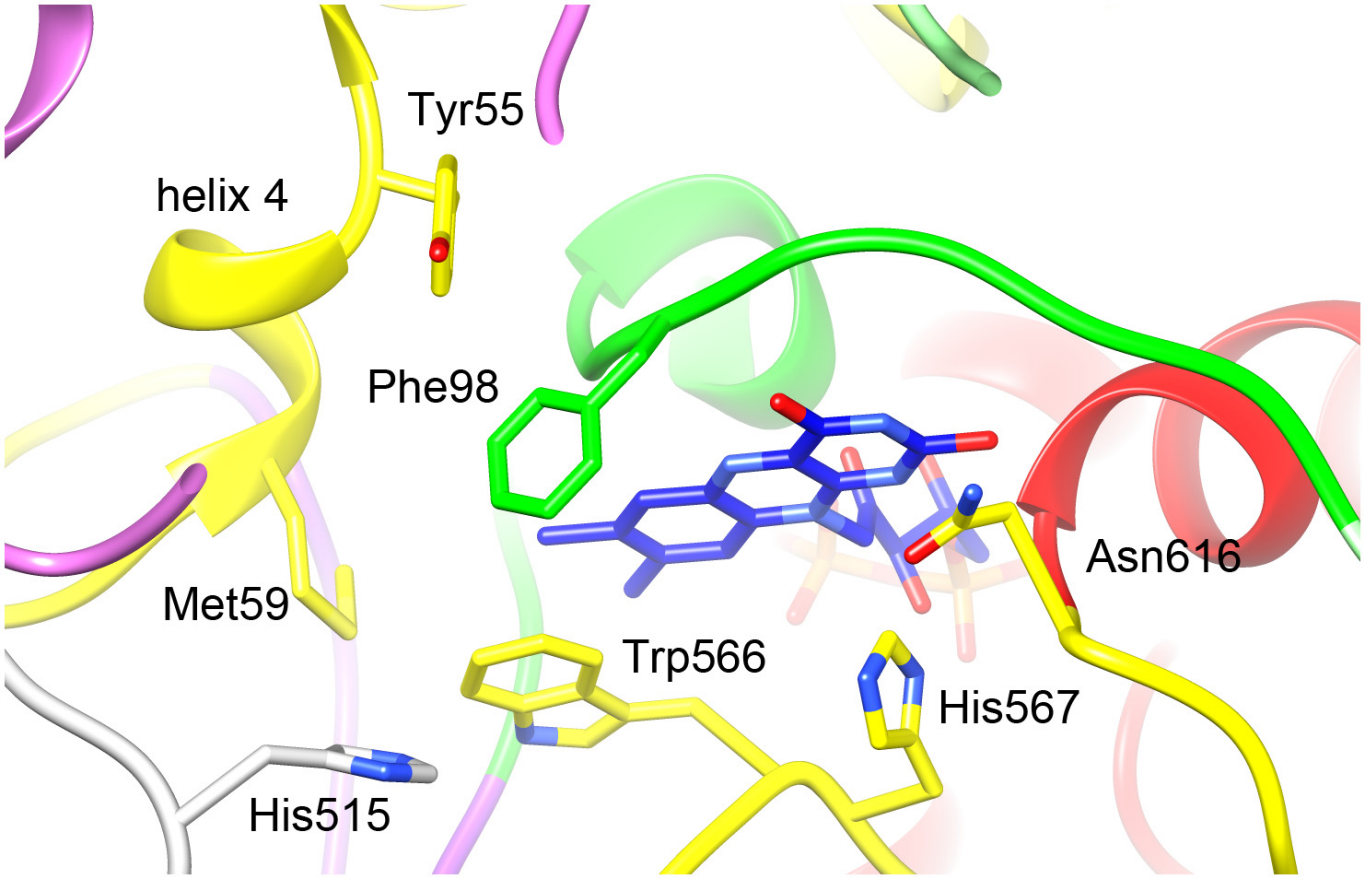
The active site. Domain colors as in Fig 5a. His567 and Asn616 play a role in catalysis. The side chains of the other indicated residues restrict substrate access to the active site and are probably involved in the specificity of AOX for small alcohols. His515 is part of a different subunit (grey).

The FAD is buried deep inside the complex in an elongated conformation as in the other GMC family enzymes (Figs 4b and 5). The adenine moiety is flanked by helix H11 and the three-stranded β-sheet D. The absolutely conserved Glu38 interacts with the ribose. The negative charge on the pyrophosphate is stabilised by helix H1, which is flanked by two β strands that are part of the five-stranded β-sheet A, a highly conserved pattern in FAD binding proteins. The C-terminal helix H25 is packed against H1 and is structurally highly conserved in the GMC family, although the sequences diverge (S1 Fig). The flavin domain of FAD is coordinated by residues on the other side of this β sheet, the flavin attachment loop (S1 Fig, Fig 4b), which is found throughout the GMC family. Asn97 interacts closely with the flavin ring; Helix H6, a short, conserved 3_10_ helix containing two or three consecutive glycine residues, interacts with the ribityl chain and appears to keep the FAD in its extended conformation (Fig 4b). The large side chain of Phe98 reduces the space available for the substrate (Fig 7); in this position there is a tyrosine in AAOX, but it is replaced by a serine in CHOX and a glycine in GOX and in cholesterol oxidase (S1 Fig).

### The active site and substrate binding domain

The active site of GMC family oxidases is neighbouring the FAD isoalloxazine ring, where there is a large cavity bounded at the bottom by β-sheet C. The reaction mechanism of the GMC family oxidases involves two half-reactions in which first a hydride is transferred from the substrate to the isoalloxazine and then the FAD is reoxidized by molecular oxygen, forming H_2_O_2_ [3, 5, 25, 47]. The active site consists minimally of the completely conserved His567 that plays a role in the oxidation of the alcohol substrate [48, 49] and Asn616, which is replaced by a histidine in some enzymes, but has been shown to have an important role in catalysis in cholesterol oxidase [50] and in CHOX [51]. Close to these residues are the side chains of Trp566 and Phe98 (see below), which are conserved in AOX, but replaced by smaller residues in the other enzymes.

While the FAD binding module of all GMC family members is highly conserved, the substrate binding domain has divergent sequences to accommodate the different substrates, although its structure is also conserved (Fig 5). The core domain (cyan in Fig 5a) shared by all structures consists of the six-stranded β-sheet C, helix H20 which covers this sheet, and the β-hairpin E between strand C4 and helix H20, which has no direct role in the substrate pocket, but seems to be the main structural element linking the substrate binding and FAD binding domain. In most GMC enzymes with the exception of cholesterol oxidase helix H24 is also part of this domain, linking H20 to the β-sheet. Variation in the substrate binding domains of the various enzymes arises due to the presence of different insert sequences. In AOX these are the tetramer helix H14 between strands C5 and C2, the dimerization helix H18 in the loop between strands C3 and C4, and the 75-residue oligomerisation loop between strand C6 and helix H24. The loop between β-strands C2 and C3 also contains inserts in several enzymes: the surface helices H16 and H17 in AOX, two longer surface helices in GOX, and a short helix in AAOX.

The sequence of helix H20 and of the β-strands in sheet C is not conserved between the different oxidases (Fig 5b), but in AOX of all species it is almost identical (S1 Fig), reflecting the role of this region in substrate specificity. Of all GMC family members AOX has the smallest preferred substrate, methanol or ethanol [4]. In our model the space available for substrate molecules near the active site is restricted by large aromatic side chains, most strikingly Trp566, Phe98 and Phe402 (Fig 7). The side chain of Trp566 is located ~4 Å away from the flavin, with the aromatic ring parallel to the isoalloxazine ring system; the other structures have a smaller aromatic side chain of tyrosine or phenylalanine in this position in the same orientation. The residue appears to play a role in FAD binding and the larger side chain in AOX may restrict the space available for substrates. Phe98 is replaced by much smaller amino acids in the other enzymes—glycine in both cholesterol oxidase and GOX, and serine in CHOX. Phe402 is part of the highly conserved AOX-specific loop between β-strands C3 and C4; however, CHOX and AAOX have a phenylalanine residue in a similar position. In cholesterol oxidase this region contains no protein, and a crystal structure [29] (pdb 1coy) shows a bound steroid substrate that occupies the space of the two phenylalanine side chains 98 and 402. In AAOX, which has aromatic alcohols as substrate and is the closest GMC family member to AOX, has aromatic residues at all three positions. In AAOX Phe502 replaces AOX-Trp556, Tyr92 AOX-Phe98, and Phe397 AOX-Phe402. The first two aromatic residues are close to the FAD and they are superposed in the structure. Phe402 and Phe397 on the other hand sit in loops which have a different structure in the two enzymes and are in slightly different positions. However, in both cases they are close enough to the other active site residues to interact. Another feature shared by AOX and AAOX but not the other enzymes is the highly restricted access to the active site. In the monomeric AAOX an insert between β-strands C3 and C4 blocks direct access to the active site [3]. In AOX a loop from another subunit containing the conserved sequence 515-HGSW appears to have the same function. The side chain of His515 from one subunit is inserted into the neighbouring protomer and is close to Trp566 (~2.3 Å) (Fig 7) and probably also to Glu563 (for which there is poor density in our map), both of which are conserved in AOX.

A possible substrate access channel leads from the tetramer interface to the FAD isoalloxazine ring, lined by hydrophobic residues, including Ala514 from the neighbouring subunit, Ile83, Pro85, Phe68, Leu61, Met59, Ile96, Phe98 and Trp566. All of these residues are conserved in AOX (only Ile83 is replaced by Val in some species), but not in the GMC family.

### The quaternary structure

Unlike the other GMC family proteins, which act as monomers or dimers, AOX forms an octameric complex, consisting of two tetramers stacked face to face. The dimensions are 130 x 130 x 105 Å. The complex is very tightly packed, with numerous interactions between subunits and only a small cavity in the center (Fig 3e, S4 Movie).

The sequence alignment of the GMC family proteins (S1 Fig) reveals several stretches unique for AOX. All are located on the periphery of the subunit (Fig 5) and most are involved in oligomerisation. In fact, almost the whole intersubunit interface is formed by AOX-specific inserts (S5 Movie).

The most significant oligomerisation module is the 75-residue insert 477–551, which is involved both in contacts within the tetrameric ring and in dimer contacts between rings. The first 50 residues form the sole contact between the two rings with extensive interactions (Fig 6b). The residues 504–521 form an exposed, uncharged extension of the monomer that is buried in a groove in the neighbouring subunit near the active site. As described above, the side chain of His515 at the end of this loop appears to be part of the substrate binding pocket (Fig 7), suggesting that the oligomerisation is essential for the catalytic activity. It has been shown that FAD-containing monomers are inactive, but can be reassembled into active oligomers [52]. Nearby, the conserved side chains of Trp518 and His561 of different monomers interact closely.

The residues 524–540 form a hairpin that extends from the monomer and interacts with the neighboring subunit in the tetramer via the loops 245–251 (an AOX-specific loop between two β strands of the FAD binding domain) and 435–440 (the loop between the two strands of beta sheet E, which is conserved in the GMC family). The last ten residues of the insert form a linker back to the protein core via the helix H24 (548–561), which is one helix turn longer than the equivalent element in other GMC family proteins.

The highly conserved AOX-specific loop 133–140 makes contact to the also AOX-specific C-terminal end of helix H10 on the top of the octamer near the fourfold axis. The four helices 335–346 (H14) form a bundle around the four-fold axis, tightly packed at its C-terminal end but with a distance of ~15 Å at the N-terminal end. The helix bundles of the two tetramers are separated by ~12 Å (Fig 6). This helix and two short flanking helices (H13 and H15) are absent in most other GMC oxidases, but present in the monomeric AAOX, where it is exposed on the surface.

**Fig 8:**
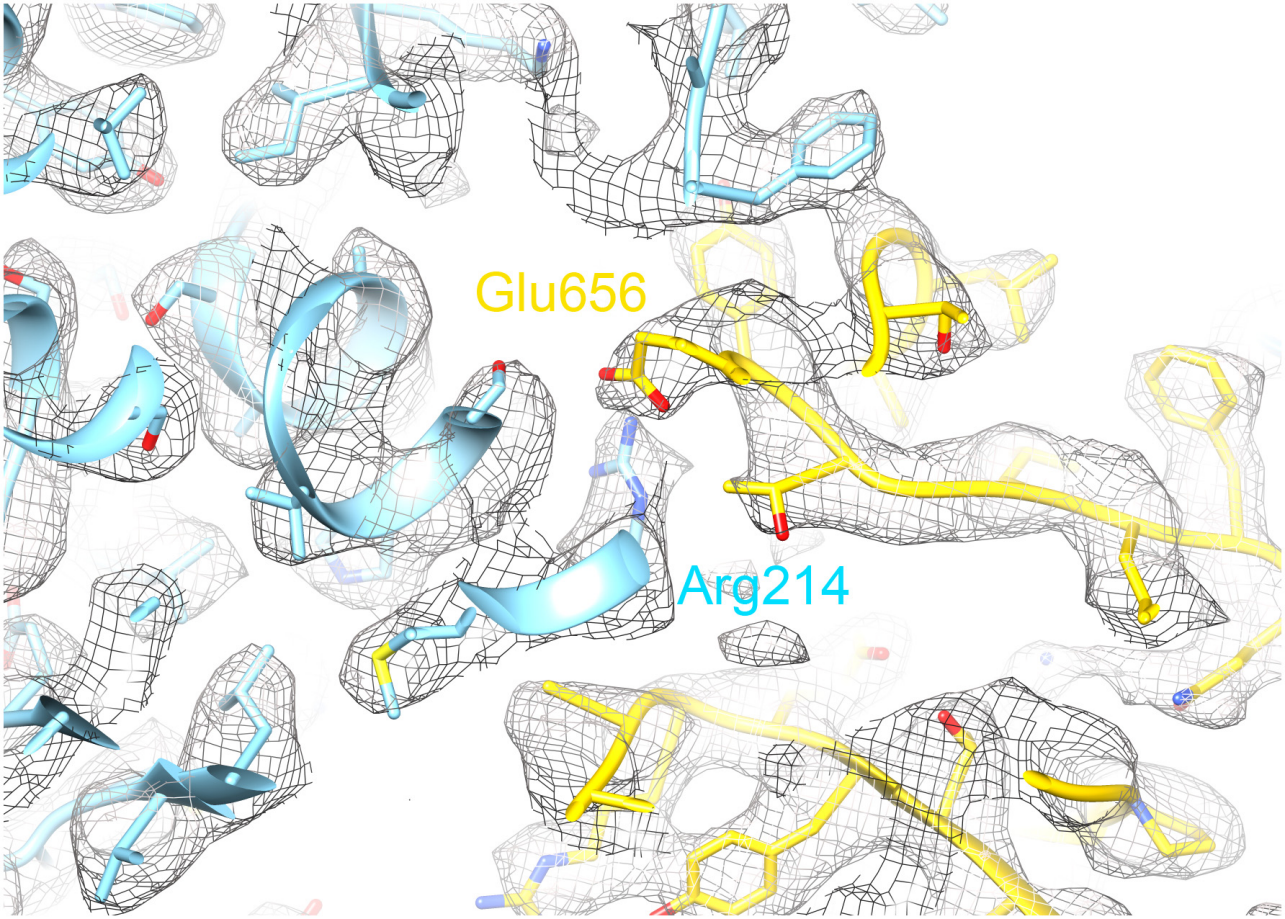
A slice of the density near the fourfold surface. A salt bridge between Glu656 and Arg214 of different subunits (gold and light blue) may contribute to intersubunit contacts.

Another tetramer contact is mediated by the C-terminal extensions 637–663, which form a ring at the surface around the fourfold axis (Fig 6). Glu656 is one of the few carboxylate side chains with good density, and it appears to make a salt bridge with Arg214 from the neighbouring residue (Fig 8). Both residues are conserved in AOX and in AOX-specific elements. The last three residues enter the cavity between monomers. The C-terminal residue, Phe663, is located just above the tetramer helix 335–346 and also forms contacts with the AOX-specific loop around residue 198 and with Asn154, also in an exclusive AOX element.

Finally, the residues 402–411, an AOX-specific extension of the loop between the β strands C3 and C4, make a dimer contact between the two tetramer rings. The only AOX-specific region not involved in intersubunit contacts is the insert between β-strands C2 and C3, two helices H16 and H17, located on the outside of the octamer. This region is not conserved and CHOX has just a short loop between the β-strands whereas GOX and AAOX each have a different helical structure (S1 Fig).

GOX occurs as a dimer. Although the two segments involved in dimer contacts [22] are conserved in AOX (the FAD covering lid between α4 and α6, and the E1-E2 β-turn) (S1 Fig), the dimer interface is completely unrelated to the AOX oligomer. Dimer formation in GOX has been linked to FAD binding, probably due to a dual role of the FAD covering lid in FAD binding and dimerization [22]. AOX oligomerisation occurs post-translationally in the peroxisome [53] and is dependent on the presence of FAD [54]. Furthermore, only octamers are active; octamers dissociate in 80% glycerol into FAD-containing but inactive monomers, and subsequently reassemble to active octamers [52]. In AOX, the dimer interface is close to the FAD isoalloxazine ring and the substrate binding pocket and the conserved side chain of His515 in the 75-residue AOX-specific oligomerisation loop (S1 Fig) appears to be part of the substrate binding pocket (Fig 7). Loss of this interaction in the monomer may be the cause of the observed inactivation of the FAD-containing monomer [52]. Helix 4, which is fully conserved in AOX but is absent in the other GMC family members, is packed tightly within the dimer and thus appears to play a role in dimerization, and also in substrate binding through the interaction of Met59 with Trp566 and with His515 of the dimer partner (Fig 7). Interestingly, in the monomeric AAOX an AAOX-specific insert between β-strands C3 and C4 blocks direct access to the active site [3], a role that in AOX is played by the loop from the dimer partner around residues 515–519. In the monomeric cholesterol oxidase (pdb 1cox) [28] the active site is covered by three hydrophobic loops, all absent in the other family members. One of these loops changes conformation upon ligand binding (pdb 1coy) [29]. In dimeric GOX [22] the active site is covered by contributions from the dimer partner, in pyranose-2-oxidase (pdb 1tt0) [24], a dimer-of-dimers homotetramer, by a loop connecting the β-strands C3 and C4 that is unique for that enzyme. Cellobiose dehydrogenase [27] has a fused N-terminal cytochrome domain that blocks substrate access. Thus all GMC oxidoreductases have found a different mode of modulating substrate specificity.

### Peroxisomal crystal formation

**Fig 9:**
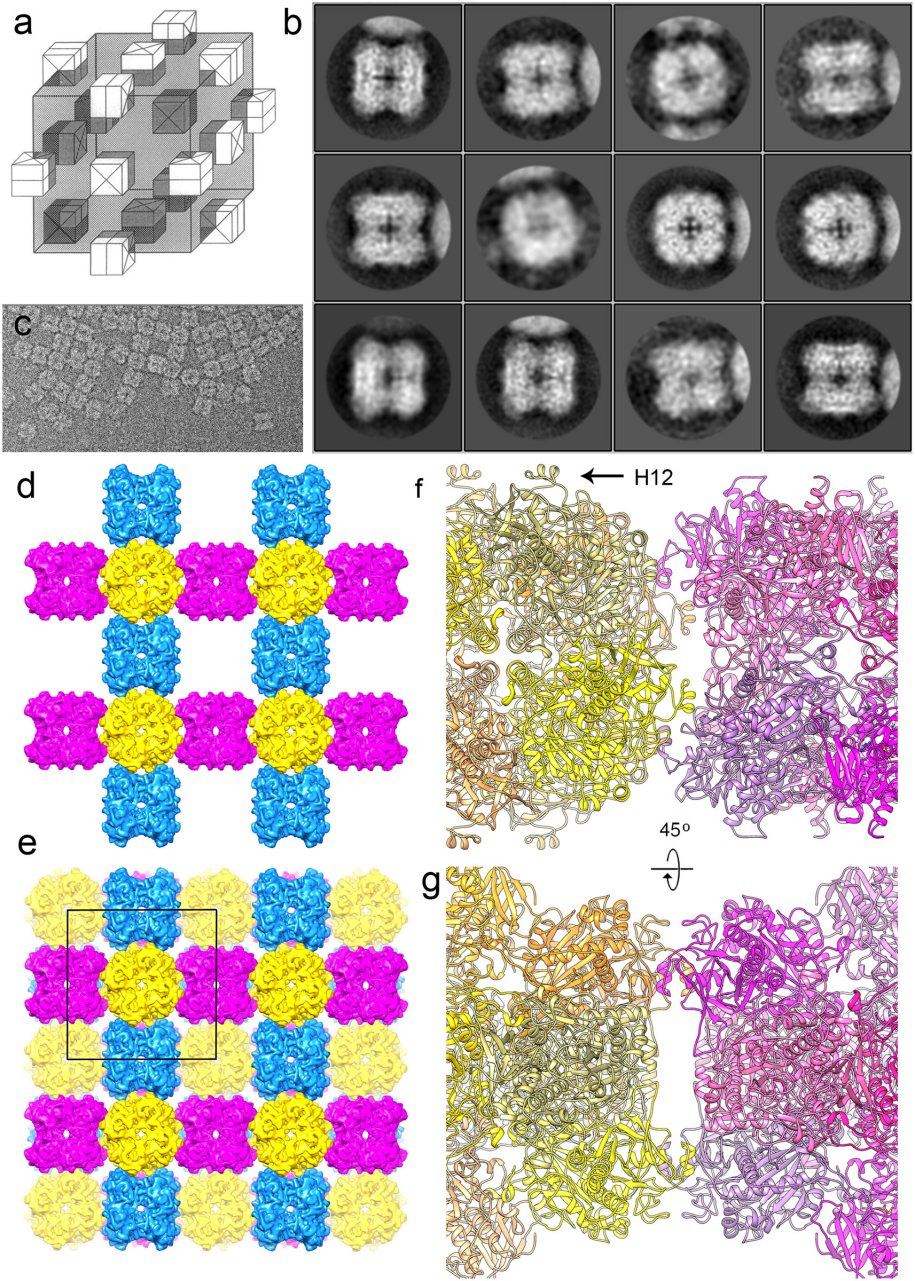
Peroxisomal AOX crystal packing. (a) Schematic representation of the peroxisomal AOX crystal packing (reprinted from [14] under a CC BY license, with permission from the American Society for Microbiology, original copyright 1992). AOX octamers are shown as square blocks with a cross indicating the fourfold axis. One unit cell of space group I432 with cell dimensions 228x228x228 Å is shown as a cube and contains 6 octamers (shaded grey). Each octamer has four neighbours, which are 90° rotated. This packing creates rows of octamers in three perpendicular directions, while the fourfold faces of six octamers line a hole (located in the center and at the eight vertices of the cube). (b) Class averages of a subset of picked particles containing a second molecule in the box. Classes showing a side view (1, 2, 4, 5, 9, 10, 11, 12) have a convex neighbour (top view) and classes showing a top view (3, 7, 8) a concave one (side view), suggesting a preferential 90° rotation between neighbours. (c) Part of a micrograph showing several AOX molecules arranged as in the crystals. Note the alternating arrangement of top view and side views. (d,e) Model of the crystal packing. The EM map was filtered to 10 Å for clarity. Neighbouring molecules are 90° rotated relative to each other and shifted 114 Å (half a unit cell). Molecules in the same orientation are shown in the same color. The yellow molecules are viewed along the fourfold axis. (d) shows one layer and (e) two layers. A unit cell is indicated in (e). (f, g) The crystal packing is mediated by helix H12. Two models are shown with each subunit in a different shade for clarity. In (f), the molecules are in the same orientation as in (d) and (e) and in (g) 45° rotated.

AOX forms crystalline inclusions in the peroxisomes. Crystals also form readily in vitro [13–15]. In the electron microscope, AOX crystals have a characteristic checkerboard appearance and it was concluded that each AOX octamer associates with four neighbours that are rotated 90° around an axis perpendicular to the fourfold axis, giving rise to a crystal with cubic symmetry of space group I432 [14] (Fig 9a). Small clusters of proteins where occasionally observed in our cryo-EM images (Fig 9c). To characterize the association between molecules in the peroxisomal crystals, images of particles with close neighbours were analysed. 3D classification in Relion of the 38,000-particle data set put 85% of the particles in one class, but the second largest class containing 3.8% of the particles showed an additional density next to the molecule. 2D classification of the images in this class (Fig 9b) confirmed that there was a specific association between the molecules. Particles showing a top view, along the fourfold axis (class 3, 7 and 8 in Fig 9b) always had a neighbour with a concave side (i.e. side view) and particles showing a side view a convex one (i.e. top view). This confirms the proposed crystal model [14] (Fig 9a). The unit cell length of the peroxisomal crystal form is 228 Å [13, 55]; thus a model of the crystal can be built by a rotation of 90° around the twofold axis and a shift of 114 Å along this axis (Fig 9d and 9e, S6 Movie). The crystal contacts are formed by helix H12, which extends from the side of the octamer (Fig 9f). There are four such contacts between each pair of molecules. Each molecule has four neighbours on the four sides. The crystal contains large, cube-shaped holes with a diameter of >100 Å faced by the fourfold surfaces of six octamers, connected by ~70 Å channels at the corners of the holes (S6 Movie). It is conceivable that the other enzymes needed for the further processing of the products of AOX, notably DHAS and catalase, are trapped in these holes during crystal formation. The products can diffuse through the channels.

In conclusion, the quality of our map has allowed us to see key features of the AOX enzyme. The residues governing substrate recognition are readily identifiable and allow for rational protein engineering whereby the specificity of the enzyme may be altered. In addition, elements responsible for the unique oligomeric structure of AOX are recognizable, as are the regions of the protein responsible for the crystallization *in vivo*. Both of these kinds of contacts are important to the survival of the organism in conditions where methanol is the only substrate because they allow extremely dense packing of the large amounts of enzyme required for methanol utilization. In addition, the structure of the crystals also appears to be adapted for maximum methanol metabolism, with subsidiary enzymes able to penetrate within the lattice.

## Materials and Methods

### Protein purification

To stimulate production of AOX, *Pichia pastoris* cells were grown overnight in medium containing 1% (w/v) methanol. Cells were harvested and broken with glass beads using a Biopec BeadBeater. The soluble material was then subjected to a two-step fractionation using ammonium sulphate. The pellet obtained after incubation with ammonium sulphate at 30% saturation was discarded and the supernatant made to 60% to precipitate the AOX. The pellet was dialysed for 2 days against 20 mM sodium phosphate and loaded onto a column of Q-Sepharose. A linear gradient up to 1M NaCl was applied and the major peak pooled and concentrated. Finally, the AOX was subjected to gel-filtration on a Superose 6 column, concentrated to 14 mg/ml and frozen in aliquots.

### Sample preparation and cryo-electron microscopy

3 μl of a 0.7 mg/ml sample was applied to freshly glow discharged Quantifoil R2/2 holey carbon grids (Quantifoil Micro Tools, Jena, Germany) that had been pretreated in chloroform for 2 hrs. The grids were blotted for 11 sec in an FEI Vitrobot plunge-freezer at 10°C and 70% humidity. Data was collected on a JEOL 3200 FSC operating at 300 kV, with the in-column energy filter operated at 20 eV. Data was recorded on a Gatan K2 back thinned direct electron detector at a nominal magnification of 30,000x with a calibrated specimen pixel size of 1.14 Å. The images were recorded in a defocus range of 0.6–2.5 μm using movie mode with 30 0.2-s frames using an electron dose of 1.7 e^-^/Å^2^/frame, resulting in a total exposure of 51 e^-^/Å^2^.

### Image processing

The 30 frames of each 6-s movie were aligned using the whole-image motion correction method described in Li et al., 2013 [35]. The first frame was discarded and the others averaged for processing. Particle picking was carried out using the semi-automatic procedure of EMAN Boxer [56], and the contrast transfer function of every image was determined using CTFFIND3 [57] in the RELION workflow [42]. An initial small dataset of ~4000 particles was aligned and classified using Imagic V [58] and selected class averages were used to build an initial model in EMAN2 [59] applying D4 symmetry. 2D and 3D classification was performed in RELION 1.3 to check data quality. Data were refined using the gold standard refinement procedure of RELION 1.3 [60, 61]. The particle polishing procedure in RELION 1.3 [43] was applied to correct particle ensemble movements and to apply a resolution-dependent frame weighting. 20 frames with an accumulated dose of 35 e^-^/Å^2^ were used for this procedure. D4 symmetry was applied in all refinements. The post-processing procedure implemented in RELION [42] was applied to the final maps for B-factor sharpening and resolution validation [44]. The local resolution of the map was estimated with the ResMap software (available at http://resmap.sourceforge.net) [45].

### Interpretation of the map and model building

Secondary structure prediction of AOX was performed with the Jpred 3 server [62]. Multiple sequence alignment was done with Clustal Omega (http://www.ebi.ac.uk/Tools/msa/clustalo). For model building, crystals structures of the homologous GMC family oxidoreductases choline oxidase (pdb 3nne) [20], glucose oxidase (pdb 1gal) [22] and aryl-alcohol oxidase (pdb 3fim) [3] were fitted to the EM map and a structure-based alignment of the sequences of these proteins with alcohol oxidase was performed. The homologous regions of AOX where then identified in the map. The protein structure was built into the 3.4-Å EM map in Coot [63] using real space refinement. Torsion angle, planar peptide, and Ramachandran restraints were applied throughout. The structure was refined using Phenix [64] followed by rounds of manual rebuilding in Coot. The model was cross-validated against overfitting by the method described [65, 66] and showed no evidence of overfitting. The final model of each subunit contains all 662 amino acid residues of the mature protein, one FAD, and one ion. It has good stereochemistry with 94.39% of residues in the most favored region of the Ramachandran plot, 5.46% in the generously allowed regions and 0.15% outliers.

Figures were made using Chimera [67].

## Supporting Information

S1 Fig Structure-based sequence alignment of alcohol oxidase and other members of the GMC family. AOX of *Candida boidinii* (Cb), *Pichia angusta* (formerly *Hansenula polymorpha*) (Pa), *Kuraishia capsulata* (Kc) and *Pichia pastoris* (this work, bold) (AOX); aryl-alcohol oxidase from *Pleurotus eryngii* (pdb 3fim) (AAOX); choline oxidase from *Arthrobacter globiformis* (pdb 3nne) (CHOX); glucose oxidase from *Aspergillus niger* (pdb 1gal) (GLOX). Residues conserved in AOX are shown in green, residues conserved in the GMC family in red. Sequence where the structure of GLOX, CHOX or AAOX differs from AOX is shown in blue. α-helices are highlighted in yellow and β-strands in grey and numbered above the sequence. Sequence not seen in the structure is shown in grey font. Domain assignments for AOX and the consensus sequence are shown below the alignment: FAD-binding domain (red); FAD covering lid (violet); extended FAD-binding domain (yellow); flavin attachment loop (sea green) and intermediate region (green); substrate-binding domain (cyan) (assignment and coloring scheme as in ref. [2]). The conserved helix forming crystal contacts in AOX is shown in orange, double underlined. Regions unique for AOX are underlined in black. Regions involved in oligomerisation are thick underlined. Fully conserved residues in the consensus sequence are in capitals, others in lowercase.
(PDF)

S1 Movie Detail of map and model. The movie shows residues 201–216 (helix H10).
(MP4)

S2 Movie The AOX monomer with structural domains colored. FAD-binding domain (red); FAD covering lid (purple); extended FAD-binding domain (yellow); flavin attachment loop (green) and intermediate region (light green); substrate-binding domain (cyan). Helix H12, which forms crystal contacts, is shown in orange. AOX-specific inserts are pink.
(MP4)

S3 Movie The AOX octamer. Each of the eight monomers is shown in a different colour; the FAD cofactors are red.
(MP4)

S4 Movie The AOX octamer map. Each subunit is shown in a different color.
(MP4)

S5 Movie The AOX octamer with domain colors shown. Colors are as in Fig 5a: FAD-binding domain (red); FAD covering lid (pink); extended FAD-binding domain (yellow); flavin attachment loop (green) and intermediate region (light green); substrate-binding domain (cyan). Helix H12, which forms crystal contacts, is shown in orange. The longest AOX-specific insert 481–548 is shown in dark blue, other AOX-specific sequences are colored purple. The tetramer interface is exclusively made by AOX-specific elements (dark blue and purple).
(MP4)

S6 Movie The peroxisomal AOX crystal. Each AOX molecule is 90° rotated relative to its neighbour. As a consequence, the octameric molecules are arranged in three perpendicular directions, each shown in a different color. This arrangement leaves large holes and channels in the crystal.
(MP4)
