# Supplementary material for: Structure of Alcohol Oxidase from Pichia pastoris by Cryo-Electron Microscopy
Source: PLoS One. 2016 Jul 26;11(7):e0159476. doi: 10.1371/journal.pone.0159476 (PMC4961394; doi:10.1371/journal.pone.0159476)
Supplement: S1 Fig — AOX of Candida boidinii (Cb), Pichia angusta (formerly Hansenula polymorpha) (Pa), Kuraishia capsulata (Kc) and Pichia pastoris (this work, bold) (AOX); aryl-alcohol oxidase from Pleurotus eryngii (pdb 3fim) (AAOX); choline oxidase from Arthrobacter globiformis (pdb 3nne) (CHOX); glucose oxidase from Aspergillus niger (pdb 1gal) (GLOX). Residues conserved in AOX are shown in green, residues conserved in the GMC family in red. Sequence where the structure of GLOX, CHOX or AAOX differs from AOX is shown in blue. α-helices are highlighted in yellow and β-strands in grey and numbered above the sequence. Sequence not seen in the structure is shown in grey font. Domain assignments for AOX and the consensus sequence are shown below the alignment: FAD-binding domain (red); FAD covering lid (violet); extended FAD-binding domain (yellow); flavin attachment loop (sea green) and intermediate region (green); substrate-binding domain (cyan) (assignment and coloring scheme as in ref. [2]). The conserved helix forming crystal contacts in AOX is shown in orange, double underlined. Regions unique for AOX are underlined in black. Regions involved in oligomerisation are thick underlined. Fully conserved residues in the consensus sequence are in capitals, others in lowercase. (PDF) [file pone.0159476.s001.pdf]

```
                                                                 βA3          H1          βA2          H2   H3
AOX Candida boidinii                                             MAIPEEFDVIVCGGGSTGCVIAGRLANVDE-NLKVLLIENGENN-LNNPWVYLPGIY 55
AOX P. angusta                                                   MAIPDEFDIIVVGGGSTGCCIAGRLANLDDQNLTVALIEGGENN-INNPWVYLPGVY 56
AOX K. capsulata                                                 MAIPDEFDIIVAGGGSAGCCIAGRLANLDP-ELKVCLIEAGENN-LNNPWVYLPGIY 55
AOX  alcohol oxidase Pichia pastoris                             MAIPEEFDILVLGGGSSGSCIAGRLANLDH-SLKVGLIEAGENN-LNNPWVYLPGIY 55
AAOX aryl-alcohol oxidase Pleurotus eryngii                          ADFDYVVVGAGNAGNVVAARLTE-DP-DVSVLVLEAGVSD-ENVLGAEAPLLA 50
CHOX choline oxidase Arthrobacter globiformis MHIDNIENLSDREFDYIVVGGGSAGAAVAARLSE-DP-AVSVALVEAGPDD-RGVPEVLQLDRW 61
GLOX glucose oxidase Aspergillus niger  SNGIEASLLTDPKDVSGRTVDYIIAGGGLTGLTTAARLTE-NP-NISVLVIESGSYESDRGPIIEDLNAY 68
                                                                         D.iv.GgG..G...A.RL.........V.liE.G.......p.......y
```

```
         H4              H5               H6(3_10)         H7                 H8     βB1
Cb    56 PRNMRLDSKTATFYNSRPSKHLNGRRAIVPQANILGGGSSINFMMYTRASASDYDDWES----EGWTTDELLPLMKKFETYQRP----------CNNRDL 141
Pa    57 PRNMRLDSKTATFYSSRPSKALNGRRAIVPCANILGGGSSINFLMYTRASASDYDDWES----EGWSTDELLPLIKKIETYQRP----------CNNRDL 142
Kc    56 PRNMQLDSKTATFYTAEPSPHLNGRRAIVPCANILGGGSSINFMMYTRGSASDYDDWET----EGWTTKDLLPLMKKTETYQRA----------CNNRDI 141
AOX   56 PRNMKLDSKTASFYTSNPSPHLNGRRAIVPCANVLGGGSSINFMMYTRGSASDYDDFQA----EGWKTKDLLPLMKKTETYQRA----------CNNPDI 141
AAOX  51 PGL-VPNSIFDWNYTTTAQAGYNGRSIAYPRGRMLGGSSSVHYMVMMRGSTEDFDRYAAVTGDEGWNWDNIQQFVRKNEMV--VPPADNHNTSGEFIPAV 147
CHOX  62 ME--LLESGYDWDYPIEPQENGN-SFMRHARAKVMGGCSSHNSCIAFWAPREDLDEWEAKYGATGWNAEAAWPLYKRLETN----------EDAGPDAPH 148
GLOX  69 GD--IFGSSVDHAYETVELATNN-QTALIRSGNGLGGSTLVNGGTWTRPHKAQVDSWETVFGNEGWNWDNVAAYSLQAERA-RAPNAKQIAAGHYFNASC 164
         .......S.....Y........N........an.lGGsss.n.....r....d.D.we.....eGW.......l.kk.E....................
```

```
             βB2          H9                      βC1                H10      βA1    βD1
Cb    142 HGFDGPIKVSFGNYTY---PQCQDFLRACETQGI----PYVDDLEDLTTSHGAEQWLKWINRDLGRRSDTAHAFIHSTMRNKENLFLLTNTKVDKVIIE- 233
Pa    143 HGFDGPIKVSFGNYTY---PTCQDFLRAAESQGI----PVVDDLEDFKTSHGAEHWLKWINRDLGRRSDSAHAYVHPTMRNKQSLFLITSTKCDKVIIE- 234
Kc    142 HGFEGPIKVSFGNYTY---DNCQDFLRAAESQGI----PVVDDLEDLVTAHGAEHWLKWINRDTGRRSDSAHAFIHSTMRNKDNLFLVTSTKIDKIIVE- 233
AOX   142 HGFEGPIKVSFGNYTY---PVCQDFLRASESQGI----PYVDDLEDLVTAHGAEHWLKWINRDTGRRSDSAHAFVHSTMRNHDNLYLICNTKVDKIIVE- 233
AAOX  148 HGTNGSVSISLPGFPT---PLDDRVLATTQEQSEEFFFNPDMGTGH---PLGISWSIASVGNG--QRSSSSTAYLRPAQ-SRPNLSVLINAQVTKLVNSG 238
CHOX  149 HGDSGPVHLMNVPPKD---PTGVALLDACEQAGI----PRAKFNTGTTVVNGANFFQINRRADG-TRSSSSVSYIHPIV-EQENFTLLTGLRARQLVFDA 239
GLOX  165 HGVNGTVHAGPRDTGDDYSPIVKALMSAVEDRGV----PTKKDF-GCGDPHGVSMFPNTLHEDQ-VRSDAAREWLLPNY-QRPNLQVLTGQYVGKVLLSQ 257
          HG..Gpv...........p.....l.a.e..q.................HG...w..........RS.sa..y.........nl.ll......k....
```

```
                 βD2           βD3     βA4      H11          H12                         βC5        H13
Cb    234 ----NGRAVAVRTVPSKPIGDSK-VSRTFKARKQIVVSCGTISSPMVLQRSGIGEPSKLRAAGVKPIVEVPGVGKNFQDHYCYFVPYRIKHDSESFDAFV 328
Pa    235 ----DGKAVAVRTVPMKPLNPKKPVSRTFRARKQIVISCGTISSPLVLQRSGIGAAHHLRSVGVKPIVDLPGVGENFQDHYCFFTPYYVKPDVPTFDDFV 330
Kc    234 ----DGRAVGVKTVPSKPLDPKNPKTRTFRARKQIVLSCGTVSSPMVLQRSGFGDPQKLRAAGVKPLVNLPGVGRNFQDHYCFFTPYRIKPHLESFDDFV 329
AOX   234 ----DGRAAAVRTVPSKPLNPKKPSHKIYRARKQIVLSCGTISSPLVLQRSGFGDPIKLRAAGVKPLVNLPGVGRNFQDHYCFFSPYRIKPQYESFDDFV 329
AAOX  239 TTNGLPAFRCVEYAEQE-----GAPTTTVCAKKEVVLSAGSVGTPILLQLSGIGDENDLSSVGIDTIVNNPSVGRNLSDHLLLPAAFFVNSN-QTFDNIF 332
CHOX  240 ----DRRCTGVDIVDSA-----FGHTHRLTARNEVVLSTGAIDTPKLLMLSGIGPAAHLAEHGIEVLVDSPGVGEHLQDHPEGVVQFEA----------- 319
GLOX  258 -NGTTPRAVGVEFGTHK------GNTHNVYAKHEVLLAAGSAVSPTILEYSGIGMKSILEPLGIDTVVDLP-VGLNLQDQTTATVRSRITSAG------- 341
                 ...V....                ....Ar.evvlsaG.i.sP.vL..SGiG....L...G...lV..P.VG.n.qDh........i.
```

```
                     H14         H15      βC2       H16                                         H17             βC3
Cb    329 SGDKEAQKSAFDQWYATGSGPLATNGIEAGVKIRPTQAELAT----------------------------ADKGFQDGWETYFENKPDKPLMHYSVISGF 400
Pa    331 RGDPVAQKAAFDQWYSNKDGPLTTNGIEAGVKIRPTEEELAT----------------------------ADEDFRRGYAEYFENKPDKPLMHYSVISGF 402
Kc    330 RGDKDVQKKVFDQWYANGTGPLATNGIEAGVKIRPTEEELAA----------------------------MDSDFREGYEEYFRNKPDKPVMHYSVISGF 401
AOX   330 RGDAEIQKRVFDQWYANGTGPLATNGIEAGVKIRPTPEELSQ----------------------------MDESFQEGYREYFEDKPDKPVMHYSIIAGF 401
AAOX  333 R-DSSEFNVDLDQWTNTRTGPLTALIANHLAWLRLPSNS-----SIFQTFP------------------------------DPAAGPNSAHWETIFSNQW 396
CHOX  320 --------------KQPMVAESTQWWEIGIFTPTED-------------------------------------------------GLDRPDLMMHYGSVP 356
GLOX  342 -----------------------AGQGQAAWFATFNETFGDYSEKAHELLNTKLEQWAEEAVARGGFHNTTALLIQYENYRDWIVNHNVAYSELFLDTAG 419
             tetramer helix           .......                 surface loop                      ...........

                   H18        βC4           βE1            βE2          H19       H20             βE6  H21
Cb    401 FGD----HTKLPPGKYMTMFHFLEYPFSRGWLHISSDDPYDS-PDFDPGFLND--DRDMWPMVWAFKKSRETARRM---ECFAGEPTPFHPHYPCDSPAR 490
Pa    403 FGD----HTKIPNGKFMTMFHFLEYPFSRGFVRITSANPYDA-PDFDPGFLND--ERDLWPMVWAYKKSRETARRM---ESFAGEVTSHHPLFKVDSPAR 492
Kc    402 FGD----HTKIAPGKYMSMFHFLEYPFSRGYVQIKSADPYAA-PTFDPGFMND--KRDMAPMIWAYKKSRETARRM---DAFAGEVTSHHPYFPYDSDAR 491
AOX   402 FGD----HTKIPPGKYMTMFHFLEYPFSRGSIHITSPDPYAA-PDFDPGFMND--ERDMAPMVWAYKKSRETARRM---DHFAGEVTSHHPLFPYSSEAR 491
AAOX  397 FHP---AIPRPDTGSFMSVTNALISPVARGDIKLATSNPFDK-PLINPQYLST--EFDIFTMIQAVKSNLRFLSGQAWADFVIRPFDPRLRD-------- 482
CHOX  357 FDMNTLRHGYPTTENGFSLTPNVTHARSRGTVRLRSRDFRDK-PMVDPRYFTDPEGHDMRVMVAGIRKAREIAAQPAMA-EWTGRELSPGVEA------- 447
GLOX  420 ---------------VASFDVWDLLPFTRGYVHILDKDPYLHHFAYDPQYFLN--ELDLLGQAAATQLARNISNSGAMQTYFAGETIPGDNL-------- 494
            dimer contact  ..s..........RG.v.i...dpy...p..dP........D....v.a.k..............F........

                   H22                                H23                    H24
Cb    491 ALEQSLEDTRKLAGPLHLTASLYHGSWSTTIGEADKHNPSHVTSSHINVYGKNIQYTEDDDKAIEKYIKEHVETTWHCLGTNSMAPLEGNKNAPEGGVLD 590
Pa    493 ARDLDLETCSAYAGPKHLTANLYHGSWTVPIDKPTPKNDFHVTSNQVQLHS-DIEYTEEDDEAIVNYIKEHTETTWHCLGTCSMAPREGSKIAPKGGVLD 591
Kc    492 ALEMDLETSKAYGGPLHLSANLAHGSWSQPIGKPSIKNADHCTSNQVGLHA-EIEYTKEDDLAIENYIKEHTETTWHCLGTNSMAPREGSKIAPHGGVLD 590
AOX   492 ALEMDLETSNAYGGPLNLSAGLAHGSWTQPLKKPTAKNEGHVTSNQVELHP-DIEYDEEDDKAIENYIREHTETTWHCLGTCSIGPREGSKIVKWGGVLD 590
AAOX  483 ---------------------------------------------------------PTDDAAIESYIRDNANTIFHPVGTASMSPRGA-----SWGVVD 520
CHOX  448 ----------------------------------------------------------QTDEELQDYIRKTHNTVYHPVGTVRMGAVED-----EMSPLD 484
GLOX  495 --------------------------------------------------------AYDADLSAWTEIPYHFRPNYHGVGTCSMMPKEM------GGVVD 533
                          oligomerisation loop                  ....D......I.....t.yH..GT..m.p.e.      ggvlD

                     βA5                      H25
Cb    591 PRLNVHGVKGLKVADLSVCPDNVGCNTFSTALTIGEKAAVLIAEDLGYSGSALDMEVPQFKLKTYEQSGAARY    663
Pa    592 ARLNVYGVQNLKVADLSVCPDNVGCNTYSTALTIGEKAATLVAEDLGYSGSDLDMTIPNFRLGTYEETGLARF    664
Kc    591 PRANVYGVKGLKVGDLSVCPDNVGCNTYTTALLIGEKIATLVAEDLGYSGDDLAMEVPQFKLGTYEKTGLARF    663
AOX   591 HRSNVYGVKGLKVGDLSVCPDNVGCNTYTTALLIGEKTATLVGEDLGYSGEALDMTVPQFKLGTYEKTGLARF    663
AAOX  521 PDLKVKGVDGLRIVDGSILPFAPNAHTQGPIYLVGKQGADLIKADQ                               566
CHOX  485 PELRVKGVTGLRVADASVMPEHVTVNPNITVMMIGERCADLIRSARAGETTTADAELSAALA               546
GLOX  534 NAARVYGVQGLRVIDGSIPPTQMSSHVMTVFYAMALKISDAILEDYASMQ                           583
          ....V.GV.gLrv.D.S..P.....n.......ig.k.a.l...e..          tetramer contact
```
